# Status and distribution of jaguarundi in Texas and Northeastern México: Making the case for extirpation and initiation of recovery in the United States

**DOI:** 10.1002/ece3.8642

**Published:** 2022-03-21

**Authors:** Jason V. Lombardi, Aaron M. Haines, G. Wesley Watts, Lonnie I. Grassman, Jan E. Janečka, Arturo Caso, Sasha Carvajal, Zachary M. Wardle, Thomas J. Yamashita, W. Chad Stasey, Aidan B. Branney, Daniel G. Scognamillo, Tyler A. Campbell, John H. Young, Michael E. Tewes

**Affiliations:** ^1^ Caesar Kleberg Wildlife Research Institute Texas A&M University‐Kingsville Kingsville Texas USA; ^2^ East Foundation San Antonio Texas USA; ^3^ 114668 Department of Environmental Affairs Texas Department of Transportation Austin Texas USA; ^4^ Present address: 14739 Department of Biology Millersville University Millersville Pennsylvania USA; ^5^ Present address: Tahoe National Forest, U.S. Forest Service Camptonville California USA; ^6^ Present address: Department of Biological Sciences Duquesne University Pittsburgh Pennsylvania USA; ^7^ Present address: Predator Conservation, AC México D.F. México; ^8^ Present address: Custer‐Gallatin National Forest U.S. Forest Service Waubay South Dakota USA

**Keywords:** camera‐trap, endangered, extirpation, felid conservation, *Puma yagouaroundi*, recovery, South Texas, Tamaulipas

## Abstract

The jaguarundi (*Puma yagouaroundi)* is a small felid with a historical range from central Argentina through southern Texas. Information on the current distribution of this reclusive species is needed to inform recovery strategies in the United States where its last record was in 1986 in Texas. From 2003 to 2021, we conducted camera‐trap surveys across southern Texas and northern Tamaulipas, México to survey for medium‐sized wild cats (i.e., ocelots [*Leopardus pardalis*], bobcats [*Lynx rufus*], and jaguarundi). After 350,366 trap nights at 685 camera sites, we did not detect jaguarundis at 16 properties or along 2 highways (1050 km^2^) in Texas. However, we recorded 126 jaguarundi photographic detections in 15,784 trap nights on 2 properties (125.3 km^2^) in the northern Sierra of Tamaulipas, Tamaulipas, México. On these properties, latency to detection was 72 trap nights, with a 0.05 probability of detection per day and 0.73 photographic event rate every 100 trap nights. Due to a lack of confirmed class I sightings (e.g., specimen, photograph) in the 18 years of this study, and no other class I observations since 1986 in the United States, we conclude that the jaguarundi is likely extirpated from the United States. Based on survey effort and results from México, we would have expected to detect jaguarundis over the course of the study if still extant in Texas. We recommend that state and federal agencies consider jaguarundis as extirpated from the United States and initiate recovery actions as mandated in the federal jaguarundi recovery plan. These recovery actions include identification of suitable habitat in Texas, identification of robust populations in México, and re‐introduction of the jaguarundi to Texas.

## Graphical abstract

From 2003 to 2021, we conducted camera‐trap surveys across southern Texas and northern Tamaulipas, México to survey for wild cats (i.e., jaguarundi, ocelots, bobcats). After 350,366 trap nights at 685 camera sites, we failed to detect jaguarundis at 16 properties and along 2 highways (1050 km^2^) in Texas, but had 126 detections in Mexico. Due to a lack of confirmed class I sightings (e.g., specimen, photograph) in the 18 years of this study, and no other class I observations or roadkill since 1986, we conclude that the jaguarundi is likely extirpated from the United States.

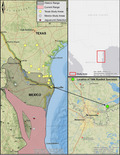

## 1 INTRODUCTION

The jaguarundi (*Puma yagouaroundi*) is an understudied, cryptic, small neotropical felid with the second‐largest distribution range of small‐bodied cats in the western hemisphere (Caso et al., 2015; Giordano, 2015). Jaguarundis arose in the Puma‐genetic lineage and share common ancestors with the much larger puma (*Puma concolor)* in North America. Jaguarundis have an atypical felid appearance, with an elongated body, short limbs, and a small oval‐sized, blunt head, thus leading to being referred to as weasel or otter cats (Giordano, 2015). Unlike the Puma, and other small cats (e.g., ocelot [*Leopardus pardalis*] and bobcat [*Lynx rufus*]) in North to South America, empirical data on current jaguarundi distribution and ecology is poorly known (Giordano, 2015; Hunter, 2015). Recent research has derived data from larger camera trapping and ecological niche modeling studies (Coronado‐Quibrera et al., 2019; Di Bitetti et al., 2010; Pereira et al., 2011; Sanchez‐Cordero et al., 2008; Santos et al., 2019). Furthermore, the most cited sources of jaguarundi ecology are derived from verified museum and citizen science occurrence data (GBIF, 2021) and gray literature including popular articles (Grassman & Tewes, 2004), ecological books, and field guides (Hunter, 2015, 2019; Schmidly & Bradley, 2016), theses and dissertations (Caso, 1994, 2013; Goodwyn, 1970), and unpublished technical reports (Bailey, 1905). Due to the lack of large‐scale studies and unknown population parameters, jaguarundis are listed as least concern by the International Union for the Conservation of Nature (IUCN; Caso et al., 2015; Hunter, 2015); however, it is considered threatened in México, and near threatened in Argentina (Caso et al., 2015). The Gulf Coast jaguarundi (*cacomitli)* is the only subspecies of jaguarundi previously confirmed in the United States and is listed as federally endangered under the U.S. Endangered Species Act (USFWS, 2013). Threats to jaguarundi include the harvest of individuals for hunting (Giordano, 2015), poultry depredation (Coronado‐Quibrera et al., 2019; Giordano, 2015), road mortality (Cerqueira et al., 2021; USFWS, 2013), and habitat loss and fragmentation associated with agriculture and urbanization (De Almeida et al., 2013; Grassman & Tewes, 2004; Tewes & Everett, 1986; USFWS, 2013).

Jaguarundis historically occurred from central Argentina and Uruguay, throughout the Amazonia Basin, north through Central America, into northeastern México and southern Texas (Caso, 2013; Caso et al., 2015; Giordano, 2015; Hunter, 2015). Today, jaguarundis are known to occur in South America and into México, however, distribution in parts of northern México and the United States is unknown (Giordano, 2015, Hunter, 2019). In the United States, jaguarundis were thought to be restricted to the southern Texas borderlands (i.e., Willacy, Cameron, Hidalgo, and Starr counties) based on class I sightings (i.e., physical specimen, photograph, DNA sample, track) (Bailey, 1905; Goodwyn, 1970; Schmidly & Bradley, 2016; Tewes & Everett, 1986). In Texas, Tewes and Everett (1986) postulated the species may have ranged north and west into the Edwards Plateau or along the mid‐coastal plain based on then credible class II sightings (i.e., unverified sightings without physical evidence or verified photographs) between 1960 and 1982. Other class II sightings have been reported in Big Bend National Park (Giordano et al., 2011), and Arizona (Giordano, 2015; Grigione et al., 2007, 2009). In northern México, class I observations exist in Tamaulipas (Caso, 1994, 2013; Caso & Domínguez, 2018; Carvajal‐Villareal, 2016), Nuevo Leon (Carvajal‐Villareal et al., 2004; Salinas‐Camarena et al., 2016), and most recently Sinaloa and extreme southern Sonora (GBIF, 2021). Other class II sightings have been reported in Durango and throughout Sonora (Brown & Lopez‐Gonzalez, 1999; Grigione et al., 2007, 2009).

Jaguarundis occur in a diverse composition of vegetation communities such as tropical and subtropical deciduous forest, swamp and savanna woodland, semi‐arid thorn forest, pine‐oak forest, and human‐altered areas such as cattle pastures (Caso, 2013; Coronado‐Quibrera et al., 2019; Giordano, 2015; Sallinas‐Camarena et al., 2016). Jaguarundis may occur in open areas with thick vegetation communities with dense herbaceous, or woody understories (de Oliveira, 1998; Giordano, 2015). In the United States and northern México, jaguarundis were and have been documented in Tamaulipan thornshrub communities, montane pine‐oak forests, tropical and subtropical deciduous forests, and mixed agricultural areas (Bailey, 1905; Caso, 2013; Caso & Domínguez, 2018; Salinas‐Camarena et al., 2016; Tewes & Everett, 1986). Jaguarundis appear to be adaptable to human‐altered environments and low to moderate levels of human disturbance (Caso et al., 2015), often occurring within 500 m of human settlements in San Luis Potosi, México (Coronado‐Quibrera et al., 2019).

In the United States, the last confirmed Class I sighting of a jaguarundi occurred in 1986 and was a road‐killed individual 3.2 km east of Brownsville, Texas on State Highway 4 (Grassman & Tewes, 2004; Tewes & Everett, 1986). In 2013, the U.S. Fish and Wildlife (USFWS) published the Gulf Coast jaguarundi recovery plan, which defined recovery and management actions to recover jaguarundis in Texas, however, stopped short of classifying jaguarundi as extirpated due to lack of peer‐reviewed scientific evidence (USFWS, 2013). The 5‐year review (USFWS, 2018) confirmed the recovery plan (USFWS, 2013) represents the most current distribution records of jaguarundis in Texas. However, current range maps issued by the USFWS’s Ecological Conservation Online System does not reflect these findings and extends current potential range throughout the lower 18 counties of southern Texas (USFWS, 2021). Over the last 7 years, the Texas Parks and Wildlife Department reclassified jaguarundis as state extirpated. Furthermore, due to their continued endangered status, federal projects are still mandated to undergo Section 7 ESA consultation with USFWS for jaguarundi, resulting in mitigation potentially being done in areas where they may not occur. Similarly, little peer‐reviewed or published occurrence data exist for jaguarundi in northeastern México (Carvajal‐Villareal et al., 2004; Caso & Domínguez, 2018; Caso et al., 2015; GBIF, 2021; Grigione et al., 2007; Salinas‐Camarena et al., 2016).

A current assessment of the presence and status of jaguarundis in southern Texas and northeastern México is needed. To address this need, we collected preliminary and final metadata from 11 past and current camera trapping studies from 2003 to 2021 in seven counties in southern Texas and from 2008 to 2010 in Nuevo Leon and Tamaulipas, northeastern México. We defined three goals: (1) assess the current distribution of jaguarundis in Texas and northeastern México; (2) provide baseline camera trap survey data such as detection probability, latency to detection, trap success in areas where jaguarundis are detected; (3) provide recommendations for informing recovery actions in the United States and northeastern México.

## 2 METHODS

**FIGURE 1:**
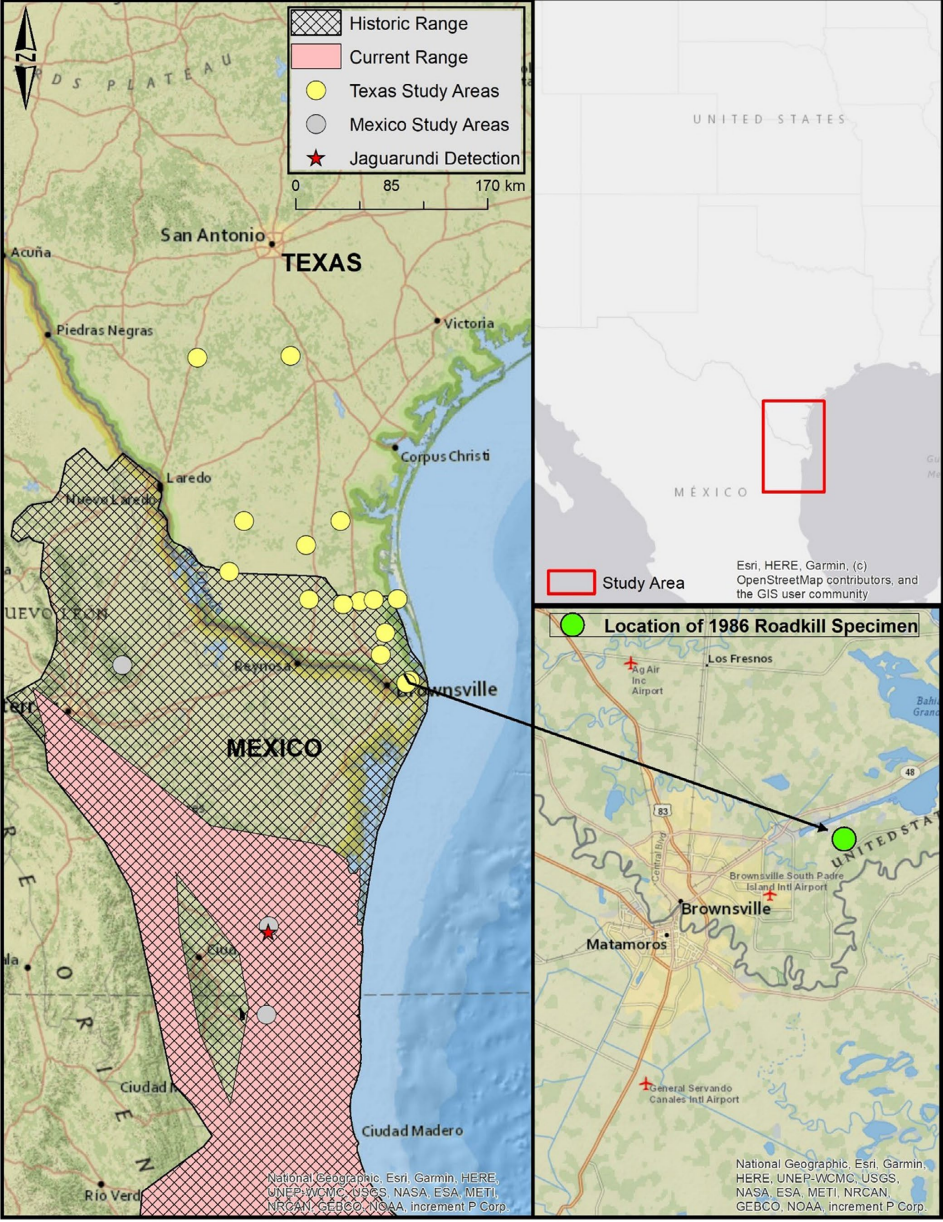
Locations of camera trap study sites in Nuevo Leon and Tamaulipas, México, and southern Texas with locations of jaguarundi detections in this study. Jaguarundi historic geographic distribution based on source maps from Goodwyn (1970) and USFWS (2013), and our suggested current geographic distribution in the region (Left Panel). Also included is the location of the last class I sighting (roadkill specimen) recovered outside of Brownsville, Texas in 1986 (bottom right panel)

In the United States, we surveyed 15 private properties and habitat patches adjacent to 2 highways; private properties included 11 ranches, 2 Nature Conservancy preserves, 1 Bureau of Reclamation area, and 1 state wildlife management unit across 10 counties in Texas (Figure 1). Surveys were conducted in the Texas–Tamaulipan thornshrub, Coastal Sand Plain, Lower Rio Grande Valley, and Lower Rio Grande Alluvial Floodplain eco‐regions (Griffith et al., 2007; Ricketts et al., 1999). Vegetation communities surveyed in Texas included deciduous (oak [*Quercus* spp.]) forests and semi‐arid Tamaulipan thornshrub communities. In México, we surveyed four private properties in the Sierra de Picachos in northeastern Nuevo Leon, two in the northern Sierra of Tamaulipas, and one in the southern part of the same Sierra in Tamaulipas (Figure 1). Survey locations occurred within the Tamaulipan Matorral, Tamaulipan Mezquital, and Veracrucian Moist Forests eco‐regions (Ricketts et al., 1999). Vegetation communities include montane deciduous forests, tropical deciduous forests, and semi‐arid thornshrub communities.

We conducted camera surveys to determine the presence of small‐ to medium‐sized felids (i.e., ocelots, bobcats, and jaguarundis) and other native carnivores in Texas (e.g., coyote [*Canis latrans*]) and México (i.e., jaguar [*Panthera onca*]) dependent on each specific study objective. In each study area, cameras were attached to wooden stakes, trees, or shrub trunks, and t‐posts approximately 0.3–1.0 m above the ground depending on terrain (Haines et al., 2006; Lombardi et al., 2020). This height range is standard worldwide for camera trapping small to large felids (Aronsen, 2010; Flores & Morales, 2019; Lombardi et al., 2020; Sollman et al., 2013; Weaver et al., 2005). Camera array designs varied by study, but camera station or individual camera placement was focused on active wildlife trails and along caliche or earthen ranch roads within or adjacent to dense to mixed woody cover patches in Texas and México. Additional camera locations in Texas included along ox‐bow lakes and highway rights‐of‐ways.

### 2.1 Period 2003–2010

We used between 7–10 active infrared Trailmaster TM1550 (Goodson and Associates, Inc., Lenexa, Kansas, U.S.) and Camtrakker passive infrared model Big Buck Surveillance System (Cam Trakker Inc., Watkinsville, Georgia, U.S.) camera traps at four properties in Texas between 2003 and 2005 (Haines et al., 2006). In Willacy County, we surveyed the Yturria‐San Francisco Ranch (Yturria Ranch; 1 October–18 December 2003) and Corbett Ranch (15 February–15 April 2004). The Yturria Ranch contained two USFWS conservation easements. In Cameron County, we surveyed The Nature Conservancy Southmost Nature Preserve and The Sabal Palm Audubon Wildlife Sanctuary (1 July–1 September 2004; Haines et al., 2006).

We used 18 active infrared Trailmaster TM1550, Camtrakker model Big Buck Surveillance System, Moultrie (Moultrie, Calera, Alabama, U.S.), and Photohunter (Photohunter, Trailtimer, St. Paul, Minnesota, U.S.) camera traps to survey the U.S. Fish and Wildlife Bureau of Reclamation's Choke Canyon Reservoir in Live Oak and McMullen counties, Texas from June 26, 2004 to September 25, 2005. We defined a grid of 0.5 km^2^ cells across the study area and each month we sampled six cells with three cameras per cell, totaling 110 sites. Cameras were checked bi‐weekly to ensure no data loss. We randomly selected 60% of camera traps to have an olfactory lure (i.e., Calvin Klein obsession for Men [Calvin Klein, New York, New York, U.S.] cologne) sprayed on carpet square attached to wooden stakes placed near each camera (Weaver et al., 2005).

We surveyed nine properties in Texas and México from 2008 to 2011. In Texas, we used 4–10 camera traps on the El Tecolote Ranch in Brooks County (30 July 2010–17 January 2011), and 9–10 camera traps on the Corbett Ranch in Willacy County (24 April 2009–10 February 2011). Cameras were rotated across 29 randomly selected locations on each ranch. In the Sierra of Picachos in northeastern Nuevo Leon, México, we surveyed the Picachos de Los Abuelos Ranch (1 May–31 August 2008), San Juan, and Los Cañones Ranches (1 Sept–30 November 2008), and La Mesa Ranch (1 December–28 February 2009). At each ranch, we set 17 WildView Extreme 4 (Stealth Cam, LLC, Grand Prairie, Texas, U.S.) camera traps spaced 300 m apart and aimed at an olfactory lure (i.e., Calvin Klein obsession for Men cologne) that was placed on a carpet square attached to a wooden stake 0.3–1 m off the ground (Weaver et al., 2005).

We surveyed Ranchos Caracol y Camotal in the northern edge of the Sierra of Tamaulipas and conducted four independent camera trap surveys in the spring (*n* = 20 camera traps; 7 February–23 May 2009), summer (*n* = 24 camera traps; 24 May–28 August 2009), fall (*n* = 58 camera traps; 29 August–17 December 2009), and winter (*n* = 30 CT; 10 December 2009–19 June 2010). Camera spacing varied from 800 to 1200 m (Carvajal‐Villareal, 2016; Stasey, 2012). We used 20 camera traps to survey Rancho San Jose de las Cañadas, located in the southern Sierra Tamaulipas for one month in June 2010 (Carvajal‐Villareal, 2016). At each camera location, we set 1–2 Cuddeback Capture 3 (Nontypical Inc, Green Bay, Wisconsin, USA) or WildView Xtreme 4 camera traps. Cameras were checked monthly, and we did not use bait or lures (Carvajal‐Villareal, 2016; Stasey, 2012).

### 2.2 Period 2010–2021

We used 20 camera traps to survey within the two Lower Rio Grande Valley National Wildlife Refuge conservation easements and the surrounding Nature Conservancy conservation easement on the Yturria‐San Francisco Ranch from April 1, 2009 to October 16, 2016. We used Cuddeback Capture and Cuddeback Color Xchange camera traps set randomly in dense thornshrub patches around the outer perimeter of the conservation easements and within a patch of live oak forest. No baits or olfactory lures were used following USFWS Permit Regulations (Permit Number permit TE822908‐0).

We conducted camera surveys on the East Foundation's El Sauz Ranch in Willacy and Kenedy counties (February 1, 2011 to April 1, 2021), San Antonio Viejo (SAV; Jim Hogg/Starr County; February 1, 2012–July 1, 2014), Santa Rosa (SR; Kenedy County; February 1, 2012–July 1, 2014), and Buena Vista Ranches (BV; Jim Hogg County; February 1, 2012–July 1, 2014). We used a 1 × 1 km grid cell between camera stations as required by USFWS (Permit Number permit TE822908‐0; Lombardi et al., 2020; Wesley Watts, 2015). We set 28 camera stations containing 2 Cuddeback^®^ Expert Scouting Cameras (February 2011–August 2016) and Cuddeback^®^ X‐Change Color and Professional White Flash cameras (August 2016–October 2020) and Reconyx PC900 (Reconyx, Holmen, Wisconsin, U.S.; October 2020–April 2021) cameras on El Sauz. Cameras were checked every four months (2011–2020) and every six months (2020–2021). We set 29, 29, and 26 camera stations on SAV, SR, and BV, respectively. Each camera station contained two Cuddeback Capture Flash cameras, which were offset 1–2 m and were monitored every three months. We used a variety of call and local scent lures on each ranch from April to July 2014 to maximize detections of bobcats (Wesley Watts, 2015). We used Feline Fix (Minnesota Trapline Products, Inc., Pennock, Minnesota, USA), Badlands Bob Gland Lure (Fur Country Lures, Jordan, Montana, USA), Cat‐Man‐Do (Milligan Brand, Chama, New Mexico, USA), and Finicky Feline #801 (Hoosier Trapper Supply, Greenwood, Indiana, USA) as local lures and Gusto (Minnesota Trapline Products, Inc.) and Snow Cat Bobcat #2 Lure (Grawe's Lures, Wahpeton, North Dakota, U.S.) as long‐distance call lures.

We conducted camera surveys on Farm‐to‐Market (FM) 1847 in Cameron County (1 September 2019–1 November 2020) and US Highway 77 in Willacy County, Texas (14 December 2020–1 May 2021) (Figure 1). Cameras on FM 1847 were placed at five planned wildlife crossing structure locations and on US Highway 77 at a newly constructed wildlife crossing structure. At each site, we used eight Reconyx HyperFire 2 camera traps, four on either side of the highway at the planned crossing location openings. On either side of the highway, one camera faced toward the road, one away from the road, and two were offset, facing north and south, respectively. This design formed a box that allowed for the identification of individual felids with unique pelage patterns. No bait or lures were used, and cameras were monitored and checked monthly.

We conducted camera surveys for carnivores on four private ranches in Hidalgo (24 camera traps; 11 February–14 June 2019), Kenedy (37 camera traps; 18 September–10 June 2021), Willacy (20 camera traps; 21 September 2020–7 January 2021), and La Salle counties (54 camera traps; 8 March–30 June 2021). We also surveyed the Texas Parks and Wildlife Department's Las Palomas Wildlife Management Area‐Arroyo Colorado Tract in Cameron County (14 camera traps: 1 September–7 November 2020). On each property, we set Browning StrikeForce Apex cameras (Browning, Arnold, Missouri, USA) or Cuddeback Professional White Flash camera traps set using the same grid structure (1 × 1 km grid) as Lombardi et al. (2020) and Wesley Watts (2015).

### 2.3 Species identification

**FIGURE 2:**
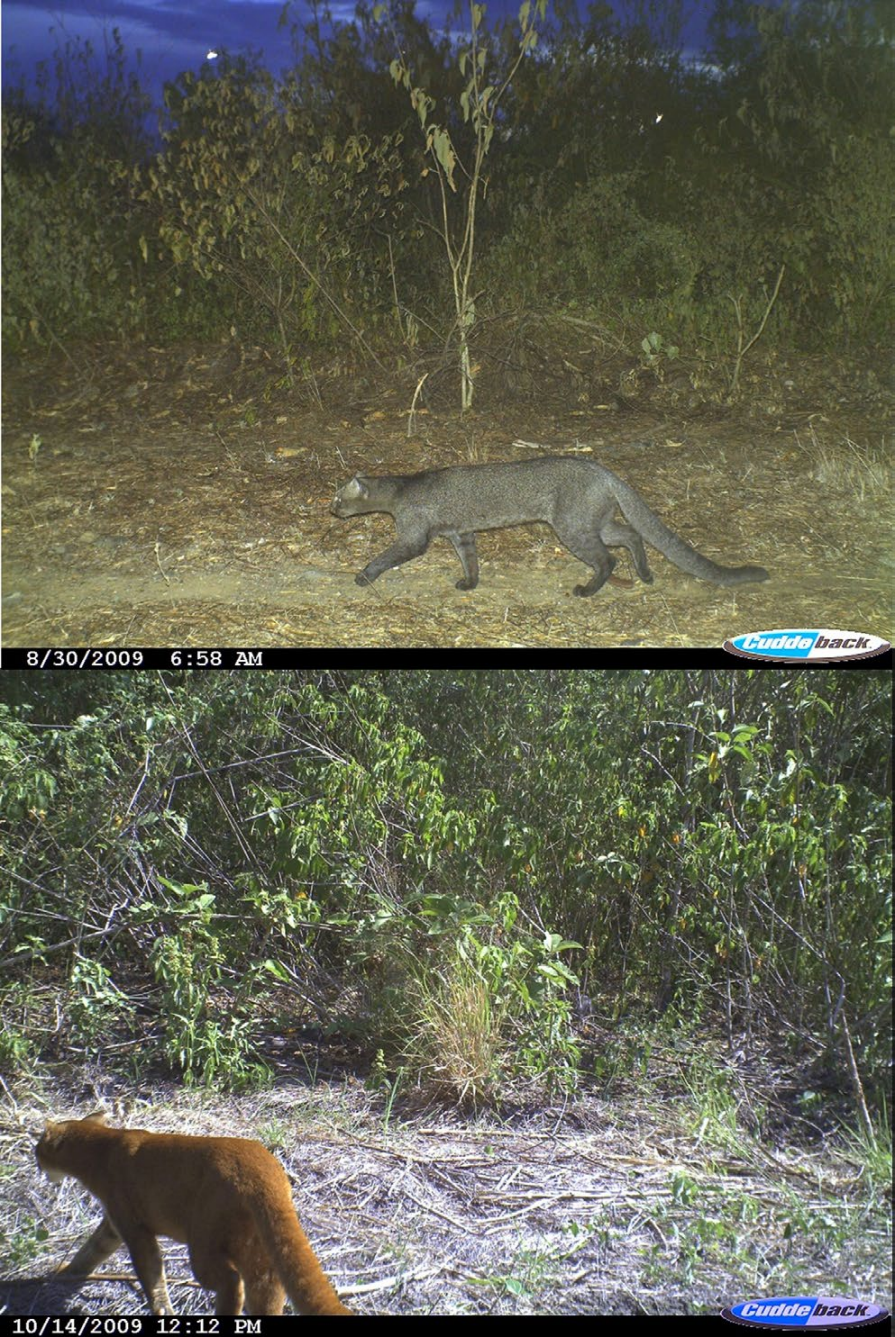
Body profile and blackish‐gray color phase of jaguarundi (top photo) and tawny‐red color phase of jaguarundi (bottom photo) on Rancho Caracol y Camotal in the Sierra of Tamaulipas in 2009

Following camera trap deployment, we classified photographs based on species and identified any jaguarundi photographs. Felids (wild and domestic) were identified by technicians and experienced biologists based on body size, morphology, and pelage patterns that are unique to each species. Any discrepancies were reviewed further with a more experienced felid biologist. Jaguarundis have a distinctive appearance with a relatively small, flat elongated blunt head profile, a long slender body (53–73.5), short legs, and a long tail (27–59 cm) (Figure 2; Caso, 2013; Giordano, 2015; Hunter, 2015). Unlike other medium‐sized felids in the study region, jaguarundis are uniformly colored with two pelage morphs: red‐brown morph ranging from bright brick red to pale tawny red, and a gray morph ranging from dark blackish gray to pale slate gray (Caso, 2013; Giordano, 2015; Hunter, 2015).

### 2.4 Analysis

Over their geographic range, there is a scarcity of basic camera trap data analyses of the jaguarundi, especially in the northern half of their distribution. In study areas where we detected jaguarundi, we conducted three camera trap analyses: latency to detection, photographic trap success, and occupancy/detection probability. We defined independent jaguarundi detections as the photographic record of a jaguarundi within a 30 min window at a specific camera station. More than one detection within a 30‐minute window at the same location was assumed to be the same individual and was not considered an independent detection (Karlin & De La Paz, 2015; Kelly & Holub, 2008). Following Kelly and Holub (2008) we determined the photographic trap success per 100 trap nights for jaguarundi by quantifying the number of detections per 100 trap nights using the formula

$${\text{TS}}_{i}=\left(\frac{N_{i}}{\sum \text{TN}}\right)\times 100$$

where TS*_i_* is trap success for jaguarundi *i*, ∑TN is the total number of trap nights over the entire survey period, and N*_i_* is the number of detections for jaguarundi *i* during the entire survey period. We quantified a crude measurement of time to the first detection by identifying the mean number of trap nights it takes to obtain the first image of the jaguarundi.

We quantified a baseline occupancy and detection rate for jaguarundi in areas where they were detected using a single‐season occupancy framework in the R package *unmarked* (Kéry & Chandler, 2012). Occupancy (*ψ*) in this context is the probability that a jaguarundi will be present at a particular site, provided it is detected, and detection (*ρ*) was defined as the probability of detecting a jaguarundi at a particular site, on a specified occasion (Kéry & Chandler, 2012). For study areas with jaguarundis, we defined a capture matrix of 1 (present) and 0 (not present) for each day. If more than one jaguarundi were detected on the same day, only the first detection was counted for that day. Because our interest was to quantify a baseline result of occupancy and detection, we did not conduct further analyses of environmental effects for each parameter. If baseline models for specific survey periods did not converge, we did not report them in this analysis.

## 3 RESULTS

In 350,366 trap nights from 2003 to 2021, we detected 2 species of felids in Texas and 6 species in México (see Dryad file). We failed to detect a jaguarundi in Texas, United States (0.00 detection events/100 trap nights). Bobcats were detected at every Texas survey location, and ocelots were only detected in the Yturria‐San Francisco Ranch (2003–2005, 2009–2016) and in the El Sauz Ranch (2011–2021). In México, we detected bobcat and puma in the Sierra of Picachos, Nuevo Leon study area. In Rancho Caracol y Camotal, northern Sierra of Tamaulipas, we documented 126 jaguarundi detections in addition to ocelot, bobcat, puma, jaguar, and margay (*Leopardus weidii*).

Jaguarundis were detected in Tamaulipan thornshrub and tropical deciduous forests. Mean photographic capture success for jaguarundis on Rancho Caracol y Camotal was 0.73 detection events/100 trap nights over 17,600 trap nights and latency to detection was three days (~72 trap nights). The baseline occupancy rate in the Sierra of Tamaulipas was 0.29, and the baseline detection probability was 0.05 detections per occasion. Overall trap success in northeastern México for jaguarundi was 0.38 detection events per 100 trap nights.

## 4 DISCUSSION

This study represents the largest combined camera trapping effort in the Texas‐México borderlands to survey for medium‐sized felids. Survey efforts in southern Texas since 2003 have failed to detect a jaguarundi, suggesting that the species is likely extirpated from Texas. During this same period, there were no additional class I observations from verified credible citizen science class I observations (GBIF), live capture (Tewes, 2019), or vehicle mortality (USFWS, 2013, Texas Department of Transportation, unpublished data) in Texas. Results from Tamaulipas, México indicate jaguarundis may be difficult to detect on camera despite a short latency to detection window. However, it suggests jaguarundis were detected in Tamaulipan Mezquital and tropical deciduous forests of the temperate Sierra Tamaulipas. These results suggest a lack of jaguarundi presence across Texas. In response, we recommend the initiation of jaguarundi recovery efforts in the United States.

We did not detect jaguarundis in their known historic habitat in southern Texas in addition to similar habitats in the region. Based on surveys in similar thornshrub communities in Tamaulipas, we would have expected to detect jaguarundi over the course of our 18‐year study if they were present on the landscape. However, the detection of ocelots, bobcats, and other small to medium‐sized carnivores (i.e., coyote, hog‐nosed skunks [*Conepatus leuconotus*]) in these habitats suggests it is not a reflection of poor‐quality habitat, rather, the absence of the species. The last confirmed specimen was obtained on state highway 4 east of Brownsville in 1986, which was only about 8–10 km north of two protected study areas along the Rio Grande we surveyed in 2003 (Haines et al., 2006). Long‐term felid live‐trapping from 1982 to 2021 in Willacy, Cameron, Hidalgo, and Kenedy counties also did not result in a jaguarundi capture (Fischer, 1998; Lombardi et al., 2021; Tewes, 2019). Furthermore, in the last 35 years, there have been no reported jaguarundi road mortalities (Texas Department of Transportation, unpub. data), but >30 road mortalities of U.S. endangered ocelots (Blackburn et al., 2020).

Other areas of Texas (i.e., Edwards Plateau, Big‐Bend‐Trans Pecos Region, and Eastern Texas coastal plain) have been postulated as a potential jaguarundi range based on credible class II sightings (Giordano et al., 2011; Tewes & Everett, 1986). Tewes and Everett (1986)’s postulated jaguarundi range may extend into the Edwards Plateau based on class II and III sightings, however, no study to date has detected jaguarundis (see Haverland & Veech, 2017; Karlin & De La Paz, 2015). Felid camera and live trap studies on the mid‐coastal plain of Texas also failed to produce class I observations (see Blankenship et al., 2006; Heilbrun et al., 2003). Like the southern Edward's Plateau and upper coastal plain, the Big Bend and Trans‐Pecos of Texas also lie outside the known historical and current geographic distribution of jaguarundis (Bailey, 1905; Caso et al., 2015). Giordano et al. (2011) assessed the credibility of jaguarundi sightings in Big Bend National Park in western Texas and found “strong support” of 40 Class II sightings (i.e., unverified sightings without physical evidence or verified photographs). However, several camera‐trap studies in the Trans‐Pecos (Hewitt, 2020), Davis Mountains (Dennison et al., 2016), Big Bend National Park (Stevens, 2017), the northern Sierra del Burro and Sierra del Carmen Mountains (Cancellare, 2018), and crowd‐sourced citizen science observations in the region have failed to document jaguarundis. Additionally, there has never been a verified jaguarundi roadkill, body parts, or tracks found in the Big Bend National Park and surrounding vicinity (R. Sikes, retired‐National Park Service, personal communication). The absence of detections or specimens from complimentary studies across the postulated extended range indicates jaguarundi presence is highly unlikely.

We documented jaguarundis in the northern edge of the temperate Sierra of Tamaulipas range, along with five other felid species (ocelot, bobcat, puma, jaguar, and margay). Our latency to detection was surprisingly low (72 trap nights), as compared to Briceno‐Mendez et al. (2017) who reported a 98.5% greater latency to detection (1140 trap nights) in Calakmul Biosphere Reserve in Campeche, México. However, detecting jaguarundis on camera was still a challenging task (0.05 detection probability per day). Overall photographic trap success for jaguarundis at this site (0.73 detection events/100 TN) was slightly greater than what was observed by Pereira et al. (2011) in Argentina (0.2 detection events/100 TN). Low photographic detection events of jaguarundis were also observed in the Atlantic Forest of Brasil (Di Bitetti et al., 2010) and Bolivian Chaco (Maffei et al., 2007). However, by comparison, camera trapping still appears to have a higher capture probability than live traps for jaguarundi (1 capture event/1320 trap‐nights; Caso, 2013). However, we do not believe low jaguarundi detection probability in México or lack of detection in Texas were due to avoidance of sympatric carnivores because jaguarundis are often documented in large multicarnivore camera trapping studies throughout their range (Di Bitetti et al., 2010; Maffei et al., 2007; Santos et al., 2019).

Based on our results, we propose northeastern México particularly in the states of Nuevo Leon and Tamaulipas, as the current northern extent of the jaguarundi's geographic range (Figure 2). While we did not detect jaguarundis in the Sierra Picachos in Nuevo Leon, other researchers have documented its presence 200 km from Texas in Nuevo Leon. Carvajal‐Villareal et al. (2004) and Salinas‐Camarena et al. (2016) documented jaguarundis in montane matorral habitat in the Parque Nacional Cumbres de Monterrey and more recently jaguarundis were detected in the Sierra Madre Oriental outside of Montemorelos (J. Tamez, unpublished data). In Tamaulipas, jaguarundis have also been documented 100–200 km south of Texas in northern Tamaulipas (GBIF, 2021), in the Sierra de San Carlos (Caso & Domínguez, 2018), and further south in coastal Tamaulipas (Caso, 1994, 2013; Giordano, 2015).

### 4.1 Conservation implications

Jaguarundis are likely extirpated in Texas, and we recommend that USFWS consider designating jaguarundis as extirpated in the United States (Texas) to move forward with jaguarundi recovery efforts as mandated in the jaguarundi recovery plan (USFWS, 2013). The length of the study (18 years) and size of the camera trapping effort (>320,000 TN in the United States) without a detection suggests jaguarundis no longer exist within; or proximate to, their historic range in southern Texas. Once designated as extirpated, we suggest that federal and state agencies follow recovery strategies as outlined in the Gulf Coast jaguarundi recovery plan. These recovery efforts include restoring, protecting, and reconnecting habitat, public outreach and education, reducing risk of road mortality, and evaluating the feasibility of jaguarundi reintroduction into South Texas (USFWS, 2013). Such efforts will require extensive and rigorous geospatial habitat models to be conducted in southern Texas to identify potentially suitable habitats for jaguarundi reintroduction. Texas is 97% privately owned and collaboration among private landowners, nongovernmental agencies, and academic institutions will be required to help ensure successful re‐introduction efforts.

We strongly recommend further population monitoring through live trapping and camera surveys in northeastern México as an integral part of the conservation and management of jaguarundis in the region. The ecology of these felids is poorly known, and new research will help fill in knowledge gaps, such as potential interactions with bobcats. Furthermore, such efforts would also aid in the identification of jaguarundi populations with robust size, density, and genetic diversity to serve as potential source populations for jaguarundi reintroduction in southern Texas.

## CONFLICTS OF INTEREST

None declared.

## AUTHOR CONTRIBUTIONS

**Jason V. Lombardi:** Conceptualization (lead); Data curation (equal); Formal analysis (lead); Funding acquisition (supporting); Investigation (equal); Methodology (equal); Project administration (supporting); Visualization (lead); Writing – original draft (lead); Writing – review & editing (lead). **Aaron M. Haines:** Data curation (equal); Funding acquisition (equal); Investigation (equal); Methodology (equal); Visualization (supporting); Writing – original draft (supporting); Writing – review & editing (supporting). **G. Wesley Watts:** Data curation (equal); Investigation (equal); Methodology (equal); Visualization (supporting); Writing – original draft (supporting); Writing – review & editing (supporting). **Lonnie I. Grassman:** Data curation (equal); Funding acquisition (equal); Investigation (equal); Methodology (equal); Writing – review & editing (supporting). **Jan E. Janečka:** Data curation (equal); Funding acquisition (equal); Investigation (equal); Methodology (equal); Visualization (supporting); Writing – review & editing (supporting). **Arturo Caso:** Data curation (equal); Funding acquisition (equal); Investigation (equal); Methodology (equal); Project administration (equal); Supervision (supporting); Writing – original draft (supporting); Writing – review & editing (supporting). **Sasha Carvajal:** Data curation (equal); Funding acquisition (supporting); Investigation (equal); Methodology (equal); Project administration (equal); Writing – review & editing (supporting). **Zachary M. Wardle:** Data curation (equal); Investigation (equal); Methodology (equal); Writing – original draft (supporting); Writing – review & editing (supporting). **Thomas J. Yamashita:** Data curation (equal); Investigation (equal); Methodology (equal); Visualization (equal); Writing – original draft (supporting); Writing – review & editing (supporting). **W. Chad Stasey:** Data curation (equal); Investigation (equal); Methodology (equal); Writing – original draft (supporting). **Aidan B. Branney:** Data curation (equal); Investigation (equal); Methodology (equal); Writing – original draft (supporting); Writing – review & editing (supporting). **Daniel G. Scognamillo:** Data curation (supporting); Investigation (supporting); Methodology (supporting); Project administration (supporting); Writing – review & editing (supporting). **Tyler A. Campbell:** Investigation (equal); Project administration (supporting); Resources (equal); Writing – review & editing (supporting). **John H. Young:** Investigation (equal); Methodology (equal); Project administration (equal); Resources (equal); Supervision (equal); Writing – review & editing (supporting). **Michael E. Tewes:** Funding acquisition (lead); Project administration (lead); Resources (lead); Supervision (lead); Writing – review & editing (supporting).

## Supporting information

Table S1

## Data Availability

Metadata used in the manuscript are available at Dryad: https://doi.org/10.5061/dryad.8sf7m0cqd
